# Capillary electrophoresis-mass spectrometry as a tool for *Caenorhabditis elegans* metabolomics research

**DOI:** 10.1007/s11306-023-02025-7

**Published:** 2023-06-23

**Authors:** Liesa Salzer, Philippe Schmitt-Kopplin, Michael Witting

**Affiliations:** 1 Research Unit Analytical BioGeoChemistry, Helmholtz Zentrum München, Neuherberg, Germany; 2 Chair of Analytical Food Chemistry, TUM School of Life Sciences, Technical University of Munich, Freising-Weihenstephan, Germany; 3 Metabolomics and Proteomics Core, Helmholtz Zentrum München, Neuherberg, Germany

**Keywords:** Metabolomics, Caenorhabditis elegans, Mass spectrometry, Capillary electrophoresis, CE-MS, HILIC-MS, daf-2

## Abstract

**Introduction:**

Polar metabolites in *Caenorhabditis elegans (C. elegans)* have predominantly been analyzed using hydrophilic interaction liquid chromatography coupled to mass spectrometry (HILIC-MS). Capillary electrophoresis coupled to mass spectrometry (CE-MS) represents another complementary analytical platform suitable for polar and charged analytes.

**Objective:**

We compared CE-MS and HILIC-MS for the analysis of a set of 60 reference standards relevant for *C. elegans* and specifically investigated the strengths of CE separation. Furthermore, we employed CE-MS as a complementary analytical approach to study polar metabolites in *C. elegans* samples, particularly in the context of longevity, in order to address a different part of its metabolome.

**Method:**

We analyzed 60 reference standards as well as metabolite extracts from *C. elegans daf-2* loss-of-function mutants and wild-type (WT) samples using HILIC-MS and CE-MS employing a Q-ToF-MS instrument.

**Results:**

CE separations showed narrower peak widths and a better linearity of the estimated response function across different concentrations which is linked to less saturation of the MS signals. Additionally, CE exhibited a distinct selectivity in the separation of compounds compared to HILIC-MS, providing complementary information for the analysis of the target compounds. Analysis of *C. elegans* metabolites of *daf-2* mutants and WT samples revealed significant alterations in shared metabolites identified through HILIC-MS, as well as the presence of distinct metabolites.

**Conclusion:**

CE-MS was successfully applied in *C. elegans* metabolomics, being able to recover known as well as identify novel putative biomarkers of longevity.

**Supplementary Information:**

The online version contains supplementary material available at 10.1007/s11306-023-02025-7.

## Introduction

The soil dwelling nematode *Caenorhabditis elegans* (*C. elegans*) is one of the premier model organisms for biomedical research introduced in 1973 by Sidney Brenner (Brenner, 2003). Recently, *C. elegans* scientists gained interest in metabolomics to study changes in metabolism during development, aging, or different disease models (Castro et al., 2013; Hastings et al., 2019; Helmer et al., 2021; Pontoizeau et al., 2014; Van Assche et al., 2015). Metabolomics is defined as the systematic measurement and (semi)quantification of metabolites in an organism, biofluid, ecosystem or others at a defined state and time point. The metabolome covers a wide range of metabolites with different polarities, molecular weights, and concentrations, many of which are still unknown (Artyukhin et al., 2018). Until now most metabolomics analyses are performed using reversed-phase liquid chromatography coupled to mass spectrometry (RPLC-MS), which is missing much of the polar fraction of the metabolome (Harrieder et al., 2022). Hydrophilic liquid interaction chromatography (HILIC) can be employed for the separation of hydrophilic metabolites, but often suffers from long analysis time and low efficiency if not optimized and used correctly (broad, non-gaussian peaks compared to RPLC). However, charged molecules are often still difficult to analyze due to strong secondary interactions with the stationary phase or undesirable interactions with the column, fittings, and metal tubing. Polar and charged metabolites can be accessed by MS hyphenation of capillary zone electrophoresis (CZE, or often simply referred to as CE), which was already applied for the metabolomics analysis of human plasma and urine, HepG2 cells, or mouse tissue (Fernández-García et al., 2020; Wild et al., 2019; Zhang et al., 2019). However, up until today and to our knowledge CE-MS has not been used in *C. elegans* metabolomics. Most of the currently known metabolites in *C. elegans* are polar and/or also contain one or more ionizable groups (Salzer & Witting, 2021), which makes them amenable to CE-MS analysis. However, we note that polar metabolites in *C. elegans* have been analyzed mainly with HILIC-MS (Beydoun et al., 2021; Gao et al., 2018; Hastings et al., 2019; Molenaars et al., 2021).

In order to enhance our understanding of metabolism in the nematode and delve deeper into unraveling its metabolome and metabolic regulation, we employed CE-MS analysis in *C. elegans* metabolomics. We compared HILIC-MS and CE-MS analysis directly on the separation of a set of 60 polar model metabolites known to be present in *C. elegans.* Our objective was to assess the orthogonality and suitability of both methods for metabolomics studies in *C. elegans*. As proof of concept, we used *daf-2(e1370)*, one of the most studied mutants in *C. elegans*, showing a prolonged lifetime compared to wild-type (WT) worms. We performed a comparative analysis using CE-MS and HILIC-MS techniques to investigate the metabolic profiles of *daf-2* mutants in comparison to WT worms. Our findings demonstrate that CE-MS represents a valuable method for *C. elegans* metabolomics because of the distinct selectivity, narrower peak widths, reduced required sample volume and increased annotation success of metabolites based on their effective mobility. Through CE-MS analysis, we were able to confirm known biomarkers and identify novel biomarkers associated with longevity in *C. elegans*, thus expanding our understanding of the molecular mechanisms underlying lifespan regulation in this model organism.

## Materials and methods

### Chemicals

Methanol (MeOH), chloroform (CHCl_3_), acetonitrile (ACN), 2-Propanol were of LC-MS grade and has been purchased from Sigma-Aldrich (Darmstadt, Germany), same as Methyl tertiary-butyl ether (MTBE, LC grade).

Ammonium acetate (5 M), ammonium formate (10 M), Bicinchoninic Acid Protein assay Kit were from Sigma-Aldrich (Steinheim, Germany), Ethylsulfate (1 mg/mL), Paracetamol (pharmaceutical primary standard) and Procaine (98%) from Sigma-Aldrich (Taufkirchen, Germany). Acetic acid and formic acid (LC-MS grade) were purchased from Fluka® Analytical (Munich, Germany). Water has been purified using a Merck Millipore Integral 3 system (18.2 MΩ; <3 ppm TOC, Millipore, Germany). Chemical reference standards (Mass Spectrometry Metabolite Library of Standards MSMLS; Bile Acid/Carnitine/Sterol Metabolite Library of Standards BACMLS) have been purchased from Sigma-Aldrich (Taufkirchen, Germany) and have been used according to their respective guidelines.

### Preparation of standard solutions

Chemical reference standards of different metabolites were dissolved in appropriate solvents at a concentration of 100 ppm (SI Table S1-S2). Mixes of metabolite reference standards were prepared containing in total 60 metabolites, each mixture containing metabolites with unique monoisotopic mass and different concentrations (50, 25, 16, 10, 5, and 1 ppm).

### *C. elegans* culturing and metabolite extraction

For metabolomics analysis, wild-type N2 Bistrol and *daf-2(e1370)* mutant worms were used and cultivated according to previously used conditions (culturing details in SI S1.2). Metabolite extraction was performed using H_2_O/MeOH/CHCl_3_ (1/3/1, v/v/v). Briefly, around 2000 worms were suspended in 500 µL extraction solvent and homogenized in a Precellys Bead Beating system (Bertin Technologies, Montigny-le-Bretonneux, France), followed by 15 min incubation in an ice-cold ultrasonic bath. The supernatant was collected and separated in two equal aliquots, in order to have the equal sample for HILIC-MS and CE-MS analysis and evaporated to dryness using a SpeedVac Savant centrifugal evaporator (Thermo Scientific, Dreieich, Germany). Dried extracts were redissolved before CE-MS analysis using 50 µL Paracetamol/Procaine/Ethylsulfate (50 ppm/10ppm/10 ppm) in H_2_O and for HILIC-MS analysis using 50 µL H_2_O/MeOH/CHCl_3_ (1/3/1, v/v/v).

### CE-MS analysis

CE-MS methods were adapted from Drouin et al. (Drouin et al., 2021). Briefly, CE separations were performed using a 7100 Capillary Electrophoresis System (Agilent Technologies, Waldbronn, Germany) equipped with an 80 cm fused silica capillary (Polymicro Technologies, Phoenix, AZ, U.S.A.) with an internal diameter of 50 µm and external diameter of 365 µM. Before initial use, the capillary was conditioned as follows: flush 5’ MeOH, 5’ water, 5’ NaOH (1 M), 5’ water, 25’ HCl (1 M), 5’ water, 5’ HCl (0.1 M), 5’ water, and 5’ 10% acetic acid in H_2_O (v/v), serving as background electrolyte (BGE). Between runs the capillary was flushed 5’ with BGE. Metabolite mixes were injected hydrodynamically at 50 mbar for 12 s (~ 13nL). *C. elegans* samples were injected at 50 mbar for 30 s (~ 32nL). Separation was performed at 25 °C and + 30 kV for cationic and − 30 kV for anionic profiling and a constant pressure of 50 mbar applied.

The CE system was hyphenated to an Agilent 6560 IM-QToF-MS (Agilent Technologies, Waldbronn, Germany) with a Dual Agilent Jet Stream ESI source via a coaxial sheath flow ESI interface using a commercial triple tube sprayer from Agilent Technologies. The sheath liquid was delivered using an Agilent 1260 Infinity Isocratic Pump with 1:100 splitter and composed of H_2_O/iPrOH/ formic acid (50/50/0.5, v/v/v) and 10 µM purine (*m/z* 121.051, [M + H] + and *m/z* 119.035 [M-H]-) and 2 µM Hexakis(1 H, 1 H, 3 H-tetrafluoropropoxy)phosphazine (HP-0921; *m/z* 922.010, [M + H] + and *m/z* 919.995 [M-H]-) as lock masses. The sheath liquid was delivered at 3 µL/min and 10 µL/min for cationic and anionic profiling, respectively. Detailed MS parameters can be found in the SI (S1.3). The MS was operated in QToF only mode and additional MS/MS spectra were acquired for *C. elegans* samples.

### HILIC-MS analysis

HILIC separation was performed on an Agilent InfinityLab Poroshell 120 HILIC-Z column PEEK-lined (150 mm ✕ 2.1 mm, 2.7 μm, 100 Å) (Hsiao et al., 2018). Anionic profiling (negative ionization mode): A: H_2_O + 10 mM ammonium acetate + 2.5 µM InfinityLab Deactivator Additive, pH = 9. B: 10% H_2_O + 90% ACN + 10 mM ammonium acetate + 2.5 µM InfinityLab Deactivator Additive, pH = 9. A nonlinear gradient was applied (see details in SI S1.4). Column temperature was 50 °C at a flow rate of 0.25 mL/min. Cationic profiling (positive ionization mode): A: H_2_O + 10 mM ammonium formate + 0.1% formic acid. B: 10% H_2_O + 90% ACN + 10 mM ammonium formate + 0.1% formic acid. Column temperature was 25 °C at a flow rate of 0.25 mL/min. Sample injection was 3 µL in both ionization modes. Hyphenation to MS was performed on the same instrument as for CE-MS. Exact MS parameters can be found in the SI (S1.4). The MS was operated in QToF only mode and additional MS/MS spectra were acquired for *C. elegans* samples.

### Data processing and statistical analysis

MS data was centroided and converted into .mzML format using MSConvert (3.0.20342) from ProteoWizard (http://proteowizard.sourceforge.net). In case of CE-MS data, the migration time scale was transformed into an effective mobility scale, using three EOF markers (SI Table S4) spiked into each sample and the R package MobilityTransformR (Salzer et al., 2022). Peak picking and evaluation of CE-MS and HILIC-MS separation of the model metabolites was performed in R using xcms (3.12.0, https://github.com/sneumann/xcms, see details in SI S1.5) (Smith et al., 2006).

Metabolomics data of *C. elegans* samples was processed with Genedata Expressionist for MSMS 13.5.4 (Genedata AG, Basel, Switzerland). Processing included chemical noise subtraction, migration time alignment, isotope clustering, peak detection, and grouping. Data was annotated in R using a workflow utilizing the MetaboAnnotation package (Rainer et al., 2022) as described below and statistical analysis was performed in Genedata Expressionist for MS Analyst module, which included normalization on the protein content and intensity drift normalization. Significantly up- and downregulated metabolites in *daf-2(e1370)* were determined using a Welch-test with a p-value < 0.05. Features were putatively annotated using an in-house developed annotation workflow based on MS^1^ and MS^2^ matching (https://github.com/michaelwitting/MetaboAnnotationGenedata). MS^2^ matching was performed against Fiehn-HILIC, MassBank, MetaboBASE, GNPS and HMDB, which were downloaded from the Massbank of North America (https://mona.fiehnlab.ucdavis.edu/) with a cosine > 0.6. The remaining features were annotated using Sirius, CSI:FingerID using the COSMIC score as additional measure of confidence (Dührkop et al., 2015; Hoffmann et al., 2022).

## Results and discussion

### Comparison of CE-MS and HILIC-MS using targeted metabolites

#### Comparison of sensitivity, relationship of peak intensities across different concentrations, variability, and peak width

**Fig. 1:**
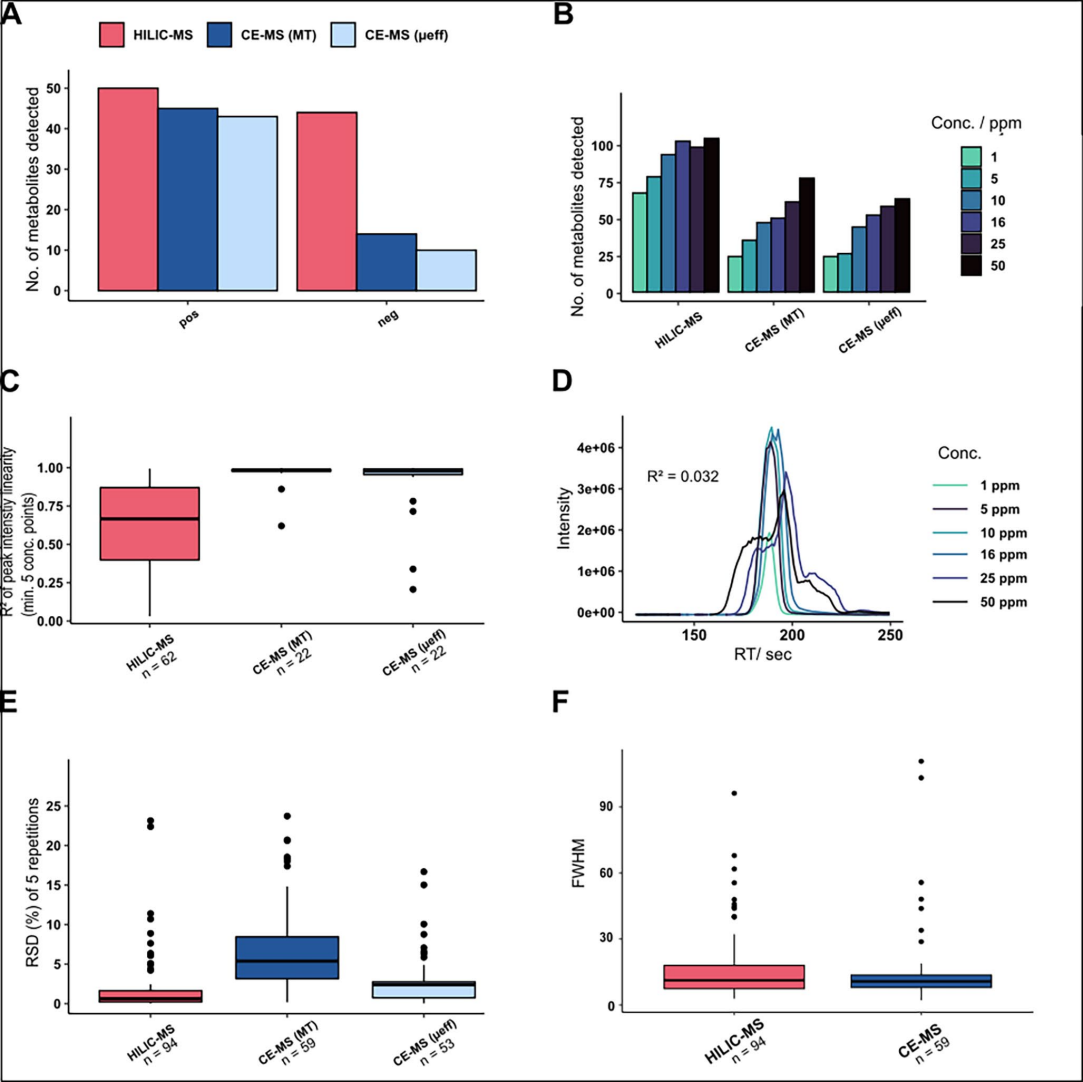
Comparison of different descriptors from CE-MS and HILIC-MS. **A**: Number of detected metabolites **B**: Minimum concentration of targeted metabolite that was detected. **C**: R^2^ of linear model build on peak-intensity relationship across different concentrations **D**: Extracted ion chromatogram (EIC) of Adenosine of HILIC-(+)-MS. **E**: Relative standard deviation (RSD) in percentage of detected metabolites. **F**: FWHM: full peak width at half maximum

We first compared both analytical setups based on a selection of 60 polar metabolites relevant for *C. elegans*. In case of CE-MS the original migration time (MT) based as well as the effective mobility (µ_eff_) scaled data were included in this comparison. In CE-MS we observed 45 in positive but only 14 metabolites in negative ionization mode (Fig. 1A). Potential reasons for low coverage in negative ionization mode was a poor ionization efficiency of the analytes due to a low quality of the electro-spray compared to positive ionization mode, also resulting in higher signal-to-noise ratios. The difference in the settings of the peak picking algorithm explains why we observe variations in the number of detected compounds between the MT scale and mobility scale of the CE-MS data (Codesido et al., 2021). In HILIC-MS we detected much more compounds (50 in positive and 44 in negative mode), even in the lowest concentration of 1 ppm (Fig. 1A and B). There are multiple potential reasons for the increased sensitivity observed in HILIC-MS. For instance, the dilution of metabolites caused by the sheath liquid-based interface, or the use of larger sample injection volumes in HILIC-MS (Kok et al., 2015; Vásconez et al., 2020) .

Next, we compared the relationship of the peak-intensity within the measured concentration range. We built a linear model of metabolites that were detected at a minimum of 5 different concentration points. In the measured concentration range, CE-MS showed a much better peak-intensity response of the linear model compared to HILIC-MS (Fig. 1C). A reason for the curvature of the peak-intensity relationship function in HILIC-MS could be saturation of the MS at higher concentrations. Moreover, peak shapes were often not good in HILIC, especially in the higher concentration range (Fig. 1D), leading to erroneous peak integration. The better response of the peak intensity indicates that we are working in CE within the linear range of the MS for most metabolites, which is necessary for proper quantification and determination of statistically significant features. If metabolite concentrations are out of the linear range of the MS, features might fall below the statistical threshold and are regarded as non-significant.

We then compared the variability of RT/ MT/ mobility of the model metabolites. The relative standard deviation (RSD) has been calculated between detected metabolites in different concentrations. As previously reported, HILIC-MS shows much more reproducible peaks then CE, having a median RSD of 0.7% across detected RT’s (Fig. 1E) (Kok et al., 2015). MT conversion into µ_eff_ shows great improvement of precision. The median RSD decreased from 5.4 to 2.4% after conversion (Figure S1). The effective mobility is constant for a substance in the same electrophoretic system (i.e. the same background electrolyte). MTs on the other hand fluctuate much more because of variations in the electroosmotic flow between runs based on difference in sample salt concentrations for example. It has also been shown that effective mobility scale transformation enhances annotation of non-targeted metabolomics data (Drouin et al., 2018; Salzer et al., 2022; Schmitt-Kopplin et al., 2020). Lastly, we compared the full peak widths at half maximum (FWHM or w_1/2_) between CE and HILIC (Fig. 1F). Our findings confirm that CE separations are highly efficient, showing very narrow and sharp peaks, due to decreased analyte diffusion compared to HILIC (Khaledi, 1998; Kohler et al., 2022). A high separation efficiency brings a high resolution which is crucial in non-targeted metabolomics analyses where we have thousands of compounds that are desired to be separated.

#### Orthogonality and complementarity of both methods

40 metabolites have been commonly detected in HILIC and CE positive and 10 in negative ionization mode (SI Table S1). We compared the RT and MT order of those metabolites (Figure S2) to show the complementarity of CE and HILIC. Since all the data points were scattered over the entire plot, it confirms orthogonal separation principles leading to no correlation between HILIC and CE. Moreover, the distinct separation principles of CE and HILIC (Figure S4) lead to the observation of different matrix effects and ion suppression during the analysis (Büscher et al., 2009), which might be advantageous for complementary detection, enhancing the discovery of statistically significant features in metabolomics studies. Moreover, reduction of the matrix effects makes it possible to load less sample compared to HILIC (13 nL vs. 3 µL).

Differences in the separation are due to the different separation mechanism, which is in HILIC more complex and includes different interactions from electrostatic, hydrophilic, and ionic effects. The separation in CE is based on the mobility of a molecule which is defined by their size and the charge as the electric field strength normalized velocity of the ions (Büscher et al., 2009; Kok et al., 2015). The complementary of CE to HILIC is a crucial assumption for the following experiments in *C. elegans* to find novel metabolites.

In summary, we observed a different selectivity, better peak-intensity response across different concentrations and narrower peak widths compared to HILIC. This is important to find potential novel biomarkers which we attempted in the next step where we applied CE-MS analysis in *C. elegans* metabolomics.

### CE-MS vs. HILIC-MS in *C. elegans* metabolomics

#### Comparison of CE-MS and HILIC-MS

In order to evaluate the usability of CE-MS for *C. elegans* metabolomics, we compared HILIC-MS and CE-MS analysis of *C. elegans* extracts of *daf-2* loss-of-function mutants and WT worms. In HILIC-MS we observed more features with a higher intensity (Table 1). In HILIC 3 µL of the sample with an approximate calculated concentration of ~ 20 worms/ µL was injected, which is equivalent to the amount of 60 worms per injection. In CE on the contrary, only 32.2 nL of the sample is injected, corresponding to a calculated value of only 0.6 worms/ injection.

**Table 1:** Number of detected and significant features and number of annotations at different msi levels

	HILIC neg	HILIC pos	CE neg	CE pos
Features	5684	5667	3534	3616
Significants	1069	1659	615	1480
Exact mass matches in the other method	69 (6,5%)	182 (11,0%)	42 (6,8%)	275 (17,4%)
msi 1	8	66	15	60
msi 2	3	18	17	39
msi 3	23	37	5	14

**Fig. 2:**
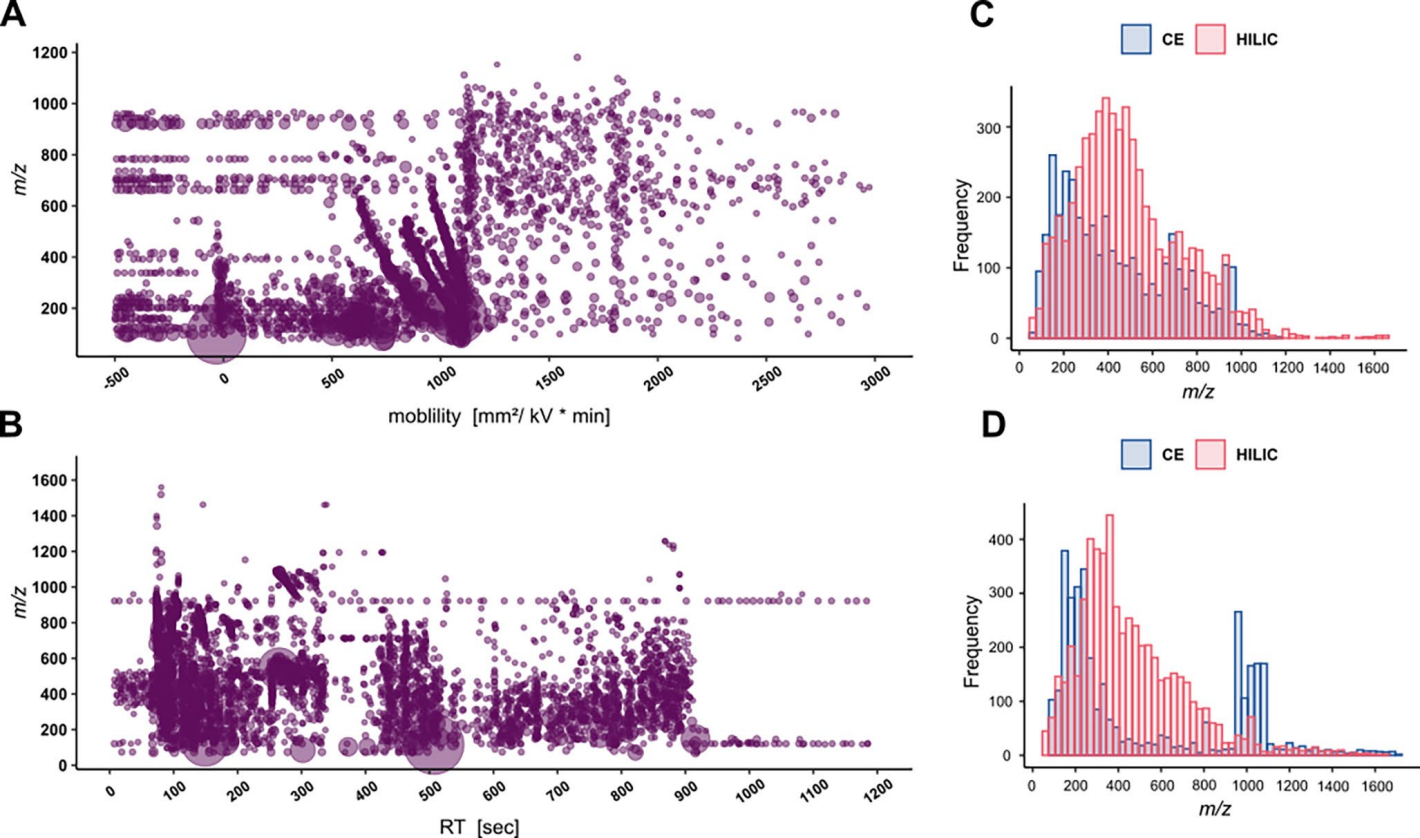
* m/z* vs. mobility/RT distribution of molecular features of a pooled quality control (QC) sample in positive ionization and separation mode at **A** CE-MS analysis and **B**: HILIC-MS analysis, size represents the relative intensity. *m/z* distribution of features of CE and HILIC in **C** positive and **D** negative ionization (and separation) mode

Comparing the *m/z* distribution of the feature tables (Fig. 2), we observe slightly more features in the lower mass range (below 250 Da) in CE-MS, which could be related to the higher electrophoretic mobility of smaller molecules. Even more, we observe an increase in the number of features in the higher *m/z* range 900–1100 Da in CE negative separation and ionization mode. We then compared significant features in CE and HILIC by exact mass matching in order to find possibly identical metabolites in both methods. Table 1 shows that more than 80% or 90% of the significant molecular features are uniquely present for each method. This number is probably even higher, since exact mass matching does not consider isomeric structures as for example the amino acids leucine, isoleucine and norleucine, having the same molecular formula (C_6_H_13_NO_2_) and therefore the identical exact mass.

Annotation is the major bottleneck in non-targeted metabolomics (Dunn et al., 2013; Mullard et al., 2015). We annotated the metabolites based on the rules of the Metabolomics Standards Initiative (MSI) in different annotation levels (Table 1)(Sumner et al., 2007). In CE-(+)-MS we had more (MSI level 1) identifications, probably due to the high separation efficiency, reduced matrix effects, and reduced ion suppression of (in HILIC) co-eluting compounds. Even with the poor spray in CE-(-)-MS we were able to identify a similar number of metabolites compared to HILIC-(-)-MS. Lower confidence annotations (MSI level 2) are based on physicochemical properties or spectral similarity matching with public MS² libraries (Sumner et al., 2007). Because the effective mobility of a molecule can be regarded as a physicochemical property in the same electrophoretic system (Rickard et al., 1991), CE gives us an increased chance of MSI level 2 annotations in combination with publicly available effective mobility libraries, such as CEUMASS (http://ceumass.eps.uspceu.es/mediator/cems_effmob_cesearch.xhtml) (Drouin et al., 2018). Indeed, our CE-MS MSI level 2 annotations were mainly based on the effective mobility library matching, highlighting the importance of effective mobility scale transformation to normalize for differences between runs and in the analytical setups.

In CE-MS we uniquely annotate 41 compounds at high certainty (MSI level 1 and 2) such as amino acid and related molecules, purines and pyrimidines, and metabolites involved in the nicotinamide pathway (i.e., nicotinamide, nicotinic acid, NAD+, tryptophan) (Johnson & Imai, 2018; Revollo et al., 2004). In HILIC-MS on the other hand we uniquely annotated 20 features i.e., different carnitines, molecules related to sugar metabolism and amino acid metabolism, purines and pyrimidines. Compounds detected exclusively in CE-MS were smaller and more polar (with lower LogP values) in comparison to HILIC-MS. This observation aligns with the overall trend of smaller *m/z* values, as depicted in Fig. 2 (Table S3).

#### CE-MS in *C. elegans* metabolomics to find new biomarkers of longevity

One of the most studied mutants in *C. elegans* is *daf-2*, which encodes for the orthologue of the insulin/ insulin-like growth factor (IGF) receptor. *daf-2* worms show prolonged lifetime compared to wild-type (WT) worms and are therefore often used to study longevity and metabolic alterations (Uno & Nishida, 2016). Mostly NMR but also HILIC-MS has been used to analyze polar metabolites in *daf-2* (Castro et al., 2013; Davies et al., 2015; Fuchs et al., 2010; Gao et al., 2018; Martin et al., 2011), showing changes in the amino acid profile (general decrease but increase in BCAAs) and carbohydrate metabolism such as an increase in trehalose, which has been directly linked to longevity (Honda et al., 2010).

**Fig. 3:**
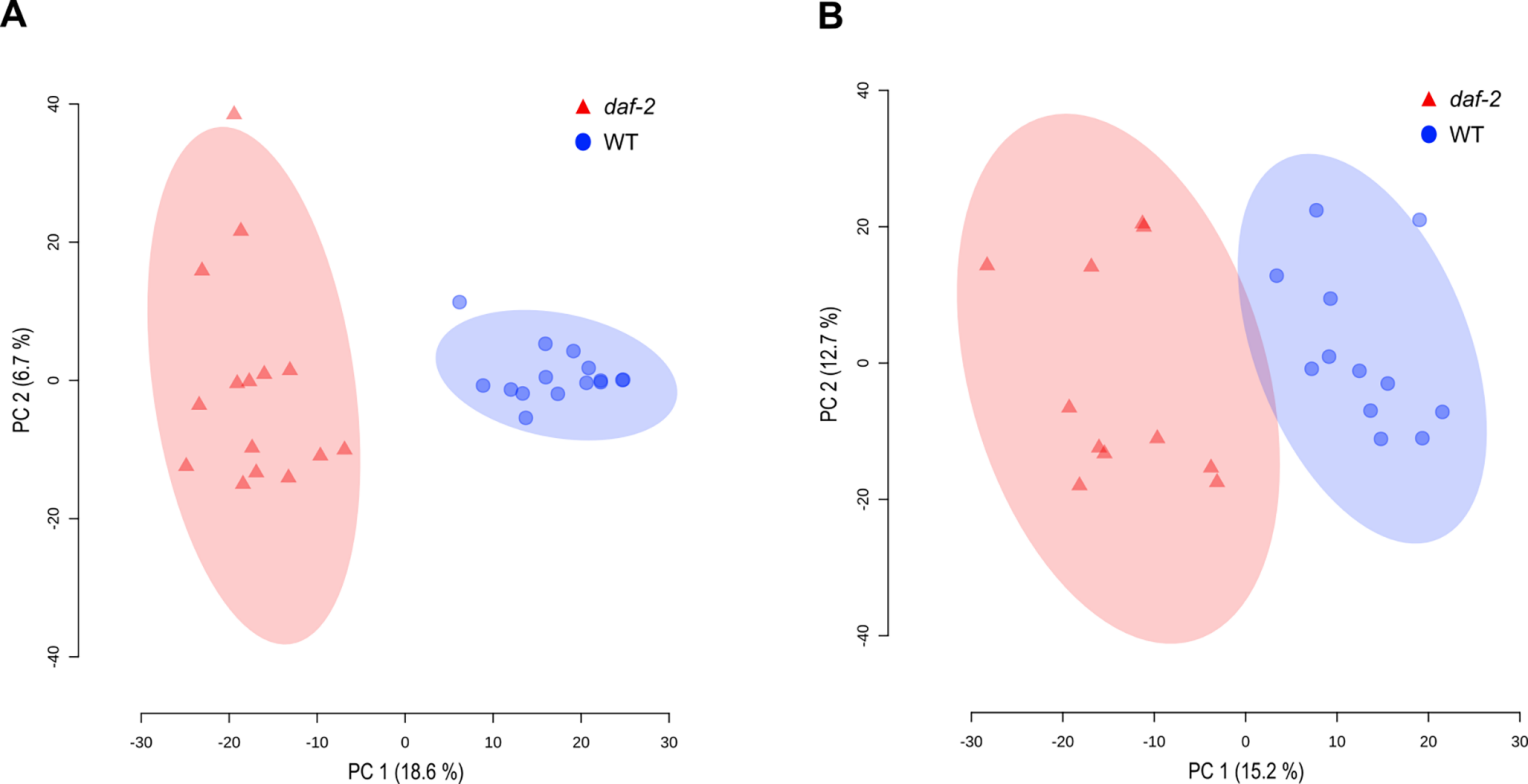
PCA scores plot **A** CE-MS positive separation and ionization mode and **B** CE-MS negative separation and ionization mode; Data was normalized on intensity drift based on QC samples, protein content, and log10 transformed

Both methods, CE-MS and HILIC-MS were able to separate the two genotypes from each other in an unsupervised principal component analysis (PCA), meaning both methods are suitable to detect molecular differences between *daf-2* and WT worms (Fig. 3).

Similarly, as previous results, our HILIC-MS analysis showed changes in the amino acid profile (upregulation in arginine, betaine, histidine, cystathionine and but decrease in tyrosine, isoleucine, alanine, kynurenine, aminoadipic acid). Beyond that, we found changes in purine metabolism intermediates as increased levels in guanine and deoxyadenosine and decreases in guanosine, uracil, and inosine. These changes suggest an alteration in energy metabolism and nucleotide salvage pathways in long lived mutants (Gao et al., 2018; Vrablik & Watts, 2013).

However, compared to literature our HILIC-MS analysis showed opposing outcomes in the levels of xanthine and adenine, which were increased in *daf-2* compared to the control group. And even more isoleucine was decreased, whereas it was found to be upregulated in *daf-2* using NMR. Divergent results of our analysis compared to the literature could be explained by differences in the *C. elegans* cultivation, such as differences in the media, food supply (strain, type and amount of bacteria), and actual age of the worm as it was shown previously for lipids (Spanier et al., 2021).

CE-MS analysis revealed similarly as in our HILIC-MS analysis an increase in uric acid, adenine, adenosine, betaine, cystathionine, phenylalanine and spermidine and a decrease in guanosine. However, different from HILIC-MS analysis, CE-MS revealed downregulation in alanine and propionyl carnitine. As already described before, 41 metabolites have been uniquely detected in CE-MS, e.g. we found changes in metabolites related in amino acid metabolism as increased levels of glycyl-proline, valine, serine, proline, N-methyl aspartic acid, trans-aconitic acid, glutamic acid, aspartic acid, taurine, N-amidino-aspartic acid and urocanic acid and downregulation in tryptophan. Moreover, CE-MS analysis showed alterations in purine and pyrimidine metabolites as hypoxanthine, paraxanthine, purine, cytidine and adenosine monophosphate. Our results in CE-MS strengthen the hypothesis of reorganization of the amino acid metabolism in long-lived worms. But different from our HILIC-MS results and from literature, most amino acids were found to be downregulated. Next, we compared metabolites that were commonly detected but were found to be significantly changed in *daf-2* in CE-MS but not in HILIC-MS. The reason why we observe discrepancies in the significant features might be the potential saturation of the MS signals. As discussed above, we observed a better peak-intensity response in CE of reference standards at different concentrations, compared to HILIC. This observation led us to postulate that there might be a potential for missing significant biological differences in a direct comparison. This hypothesis was subsequently confirmed. For example, the metabolites AMP, glutamic acid, guanidinosuccinic acid, N-acetylgalactosamine / N-acetylmannosamine, N-acetylserine, proline, serine and valine were significantly changed in CE but not in HILIC.

Ion suppression due to matrix effects can also influence the significance of features. Proline for example had a retention time of 791 s. In order to get a first impression of the matrix effect or co-eluting compounds, we determined the number of compounds eluting in the RT window 786–796 s. Indeed, our feature table revealed that within this RT range 87 features have been detected. The determined effective mobility of proline was 480 mm²/kV*min and in the range of 450–510 mm²/kV*min only 49 further features have been detected, strengthening the hypothesis of reduced ionization efficiency of e.g. proline due to ion suppression of co-eluting compounds. This effect could explain why it is not significant in our HILIC-MS analysis, whereas it was in CE-MS.

The BCAAs (leucine, isoleucine, and valine) have been reported as key metabolites significantly upregulated in *daf-2* mutants (Castro et al., 2013; Depuydt et al., 2014). Our HILIC-MS separation was able to detect and separate valine, leucine and isoleucine. However, changes in valine and leucine were not significant and isoleucine was found to be decreased in *daf-2*, questioning the reliability of the method. In our CE-MS analysis, we failed to detect leucine, but isoleucine and valine were detected and upregulated in *daf-2* mutants, which is consistent with previous findings (Castro et al., 2013; Depuydt et al., 2014). Nonetheless, these previous findings rely on NMR, which, compared to MS methods, do not suffer from ion suppression. This example shows the benefit of CE-MS and that it was possible to find the important *daf-2* longevity markers, whereas our HILIC-MS method was not capable of.

Using CE-MS, we observed different metabolite features that have been significantly changed in the long-lived mutant, including increase in most amino acids and related metabolites such as glutamic acid, guanidinosuccinic acid, aspartic acid, proline, serine, or urocanic acid, which is consistent with the literature indicating increased protein catabolism or decrease in protein anabolism and decrease in amino acid dependent energy expenditure (Depuydt et al., 2014, 2016). Also, purine salvage intermediates were upregulated in *daf-2*, i.e., AMP, which already has been reported and uridine, reported for the first time. For the first time also a decrease in hypoxanthine has been detected using CE-MS, strengthening the hypothesis of downregulation of purine degradation intermediates of long lived worms, which means altered regulation of nucleotide metabolism (Gao et al., 2018). Lastly, we also found an increase in nicotinamide, which is the precursor for the coenzymes NAD + and NADP + which are required in many biological pathways and decrease in tryptophan in *daf-2* pointing to an altered tryptophan-nicotinamide pathway. Interestingly, higher levels of nicotinamide and related molecules (nicotinic acid, NAD+) have already been linked to longevity in *C. elegans* but here, for the first time in *daf-2* (Yang et al., 2020).

## Conclusions

In this study we systematically evaluated the usability of CE-MS as tool in *C. elegans* metabolomics as a complementary platform to HILIC-MS to analyze polar metabolites. In conclusion, CE separations showed narrower peak widths, a better peak-intensity response of different concentrations pointing to less saturation of MS signals, and a different selectivity compared to HILIC-MS, making it amenable to a different part of the polar metabolome. Researchers have successfully enhanced the sensitivity of CE-MS through the utilization of innovative techniques such as nanoflow sheath liquid or sheath-less coupling approaches (Höcker et al., 2018; Moini, 2007; Schlecht et al., 2021).

Metabolomics analysis of *C. elegans daf-2* mutants revealed differences in detected metabolites between CE-MS and HILIC-MS. Even more, for some metabolites that were commonly detected the significance changed due to the poor peak-intensity response of those compounds in HILIC-MS. We annotated 41 metabolites uniquely in CE-MS, among others novel metabolite features that have been significantly changed in *daf-2* such as upregulation of purine salvage intermediates such as uridine and downregulation of purine degradation intermediates such as hypoxanthine. Lastly, we also found an altered tryptophan-nicotinamide pathway in *daf-2* which gives us new insights into understanding the mechanism of longevity in *C. elegans*.

## Electronic supplementary material

Below is the link to the electronic supplementary material.

Supplementary Material 1

Supplementary Material 2

## Data Availability

Data are currently in curation in the open-repository Metabolights under MTBLS6440.
