# Supplementary material for: Capillary electrophoresis-mass spectrometry as a tool for Caenorhabditis elegans metabolomics research
Source: Metabolomics. 2023 Jun 23;19(7):61. doi: 10.1007/s11306-023-02025-7 (PMC10289983; doi:10.1007/s11306-023-02025-7)
Supplement: Supplementary file 2 — Supplementary Material 2 [file 11306_2023_2025_MOESM2_ESM.docx]

**Supporting Information –**

**Capillary Electrophoresis - Mass Spectrometry as tool for *Caenorhabditis elegans* metabolomics research**

Liesa Salzer^1^, Philippe Schmitt-Kopplin^1,2^, Michael Witting^2,3,^*

^1^ Research Unit Analytical BioGeoChemistry, Helmholtz Zentrum München

^2^ Chair of Analytical Food Chemistry, TUM School of Life Sciences, Technical University of Munich

^3^ Metabolomics and Proteomics Core, Helmholtz Zentrum München

**S1 Methods**

*S1.1 Chemicals*

Yeast extract for use in microbial growth medium, Tryptone, NaCl, Bacteriological agar, Cholesterol, CaCl_2_, MgSO_4_, K_2_HPO_4_, KH_2_PO_4_, Methanol (LC-MS grade), Methyl tertiary-butyl ether (MTBE, LC grade), 2-Propanol (LC-MS grade), NaOH, ACN (LC-MS grade), ammonium acetate, ammonium formiate, Bicinchoninic Acid Protein assay Kit, Ethylsulfate, Paracetamol and Procaine were purchased from Sigma-Aldrich (Darmstadt, Germany). Acetic acid and formic acid were purchased from from Fluka® Analytical (Munich, Germany) and Bacto Peptone from BD Biosciences

*S1.2 C. elegans culturing*

For metabolomics analysis wild type N2 Bistrol and *daf-2(e1370)* mutant worms were used. Approximately 500 age-synchronized L1 worms were grown on NGM agar seeded with *Escherichia coli*(*E. coli)* (OP50) at 20 °C and harvested at young adult stage.

Overnight cultures of *E. coli* (OP50) were grown at 36 °C in autoclaved liquid LB-medium composed of 5 g/L yeast extract, 10 g/L tryptone and 0.5 g/L NaCl in milliQ at pH 7.0. 100 µL of *E. coli* were spread on 10 cm NGM plates that were prepared using 3g/L NaCl, 2.5 g/L Bacto Peptone, 20g/L Agar,5 µg/mL Cholesterol, 1 mM CaCl_2_, 1mM MgSO_2_, 25mM PO_4_^3-^ in milliQ water

Worms were washed from NMG plates using M9 buffer, which was 22 mM KH_2_PO_4_, 42 mM Na_2_HPO_4_, 86 mM CaCl_2_ and 1mM MgSO_4_.

*S1.3 CE-MS method*

Agilent MassHunter version B.09.00 was used for controlling and monitoring the instrument.

Source parameters were optimized in positive and negative ionization mode separately. Briefly, cationic profiling was performed at 10 psi Nebulizer gas, gas flow at 8 L/min at 300°C and 3.5 L/min Sheath Gas at 195 °C.  Fragmentor voltage was 400 V, Skimmer 65 V, Octopole 800 V, capillary 2000 V and Nozzle 2000 V.

Anions were analyzed using 10 psi Nebulizer gas, gas flow at 5 L/min at 130 °C and 3.5 L/min Sheath Gas at 140 °C.  Fragmentor voltage was 400 V, Skimmer 65 V, Octopole 800 V, capillary 2000 V and Nozzle 1800 V.

QToF only MS acquisition was performed at *m/z* 50 - 950 for metabolite mixes and 50 - 1700 for *C. elegans* samples at 2 Hz.

Worm extracts were additionally acquired in MS/MS mode with MS/MS *m/z* range 50 - 1700, MS/MS scan rate of 10 Hz, narrow isolation window and three fixed collision energies of 10, 20, and 40 eV, and max. precursors per cycle were 3 with min of 100 counts intensity.

*S1.4 HILIC-MS method*

| Column | Agilent InfinityLab Poroshell 120 HILIC-Z column PEEK-lined (150 mm ✕ 2.1 mm, 2.7 µm, 100 Å) | |
| --- | --- | --- |
|  | **Cationic Profiling**  **(Positive ionization mode)** | **Anionic Profiling**  **(Negative ionization mode)** |
| Solvent | **A:** H_2_O + 10 mM ammonium formate + 0.1 % formic acid  **B:** 10 % H_2_O + 90 % ACN + 10 mM ammonium formate + 0.1 % formic acid | **A:** H_2_O + 10 mM ammonium acetate + 2.5 µM InfinityLab Deactivator Additive, pH = 9  **B:** 10 % H_2_O + 90 % ACN + 10 mM ammonium acetate + 2.5 µM InfinityLab Deactivator Additive, pH = 9 |
| Gradient | \| **Time (min)** \| **% A** \| **% B** \| \| --- \| --- \| --- \| \| **0** \| 2 \| 98 \| \| **3** \| 2 \| 98 \| \| **11** \| 30 \| 70 \| \| **12** \| 40 \| 60 \| \| **16** \| 95 \| 5 \| \| **18** \| 95 \| 5 \| \| **19** \| 2 \| 98 \| \| **20** \| 2 \| 98 \| | \| **Time (min)** \| **% A** \| **% B** \| \| --- \| --- \| --- \| \| **0** \| 4 \| 96 \| \| **2** \| 4 \| 96 \| \| **5.5** \| 12 \| 88 \| \| **8.5** \| 12 \| 88 \| \| **9** \| 14 \| 86 \| \| **14** \| 14 \| 86 \| \| **17** \| 18 \| 82 \| \| **23** \| 35 \| 65 \| \| **24** \| 35 \| 65 \| \| **24.5** \| 4 \| 96 \| \| **26** \| 4 \| 96 \| |
| Post Time | 4 min | 3 min |
| Column Temperature | 25 °C | 50 °C |
| Flow Rate | 0.25 mL/min | |
| Injection Volume | 3 µL | |

QToF only MS acquisition was performed at *m/z* 50 - 950 at 1 Hz. Worm extracts were acquired in MS/MS mode with MS/MS *m/z* range 50 - 1700.

For positive ionization mode source parameters were 40 psi Nebulizer gas, gas flow at 5 L/min at 225 °C and 10 L/min Sheath Gas at 225 °C.  Fragmentor voltage was 400 V, Skimmer 65 V, Octopole 750 V, capillary 3000 V and Nozzle 0 V. Anionic profiling was performed using 35 psi Nebulizer gas, gas flow at 13 L/min at 225 °C and 12 L/min Sheath Gas at 350 °C.  Fragmentor voltage was 400 V, Skimmer 65 V, Octopole 750 V, capillary 3500 V and Nozzle 0 V.

*S1.5 CentWave Parameters*

|  | **HILIC** | **CE - MT** | **CE - mobility** |
| --- | --- | --- | --- |
| peakwidth | c(10, 200) | c(10, 150) | c(10, 300) |
| noise | 2000 | 1000 | 2000 (neg:1600) |
| snthresh | 20 | 3 | 20 |
| Ppm | 30 | 10 | 30 |
| Mzdiff |  | 0,01 |  |
| Prefilter | c(4, 1000) | c(5, 1000) | c(4, 500) |
| integrate | 2 | 1 | 2 |

*S1.6 Compare physicochemical properties of C. elegans metabolites*

In order to investigate the underlying base of the differences in the annotated features, we compared different molecular descriptors such as LogP, number of the longest chain, number of rotational bonds, number of bonds, number of aromatic bonds, number of aromatic atoms, number of hydrogen-bridge donors and acceptors, the topological polar surface area, the sum of the atomic polarizabilities, molecular weight, and number of basic and acidic groups.

To calculate the molecular descriptors we used the rcdk R package and the SMILES of the molecules as the respective pH (pH 9 for HILIC neg and pH 2 for CE and HILIC pos; SMILES generated with MarvinSketch).

See Table S3

**S2 Figures**


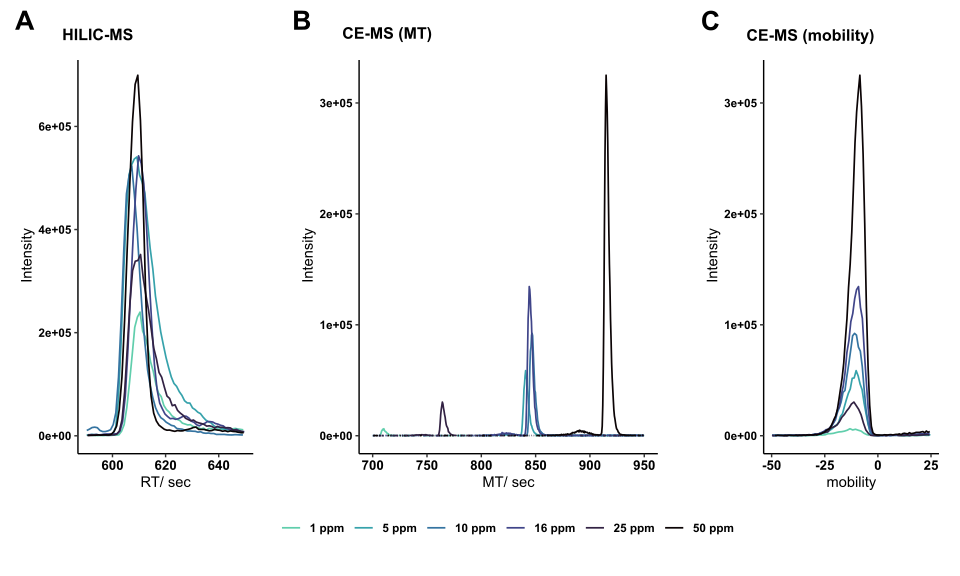


*Figure S1.* Extracted ion chromatogram (EIC)/ extracted ion electropherogram (EIE) of adenosine monophosphate (AMP) in different concentrations (1-50 ppm), from left to right: HILIC, CE (MT scale), CE (mobility scale).


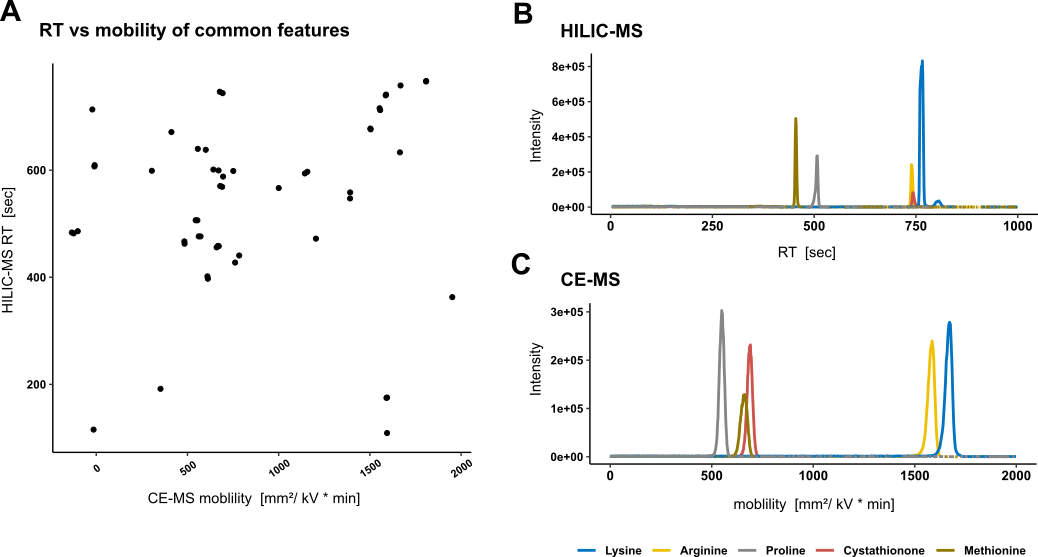


*Figure S2.* **A:** Retention time vs effective mobility of commonly detected model metabolites **B:** EIC of selected model metabolites **C**: EIE of selected model metabolites

*
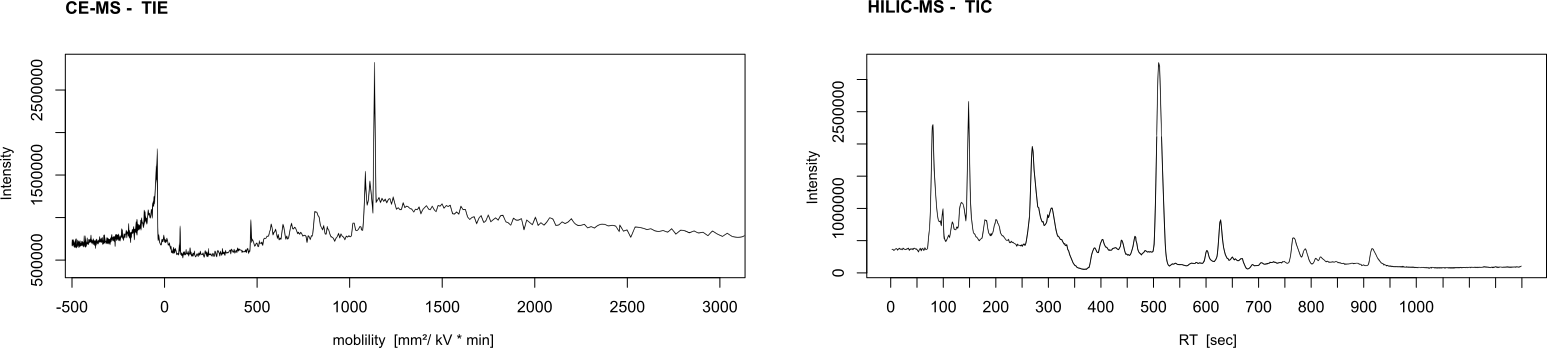
*

*Figure S3.* Total ion electropherogram (TIE) and total ion chromatogram (TIC) of QC sample measures in positive ionization (and separation) mode in CE-MS and HILIC MS


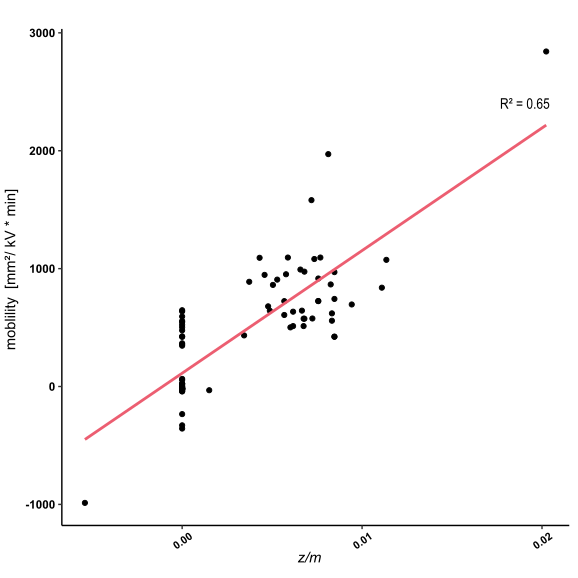


*Figure S4.* Separation principle of CE analysis demonstrated by the mobility vs. charge-to-mass ratio (*z/m*) of the metabolites annotated in *C. elegans*


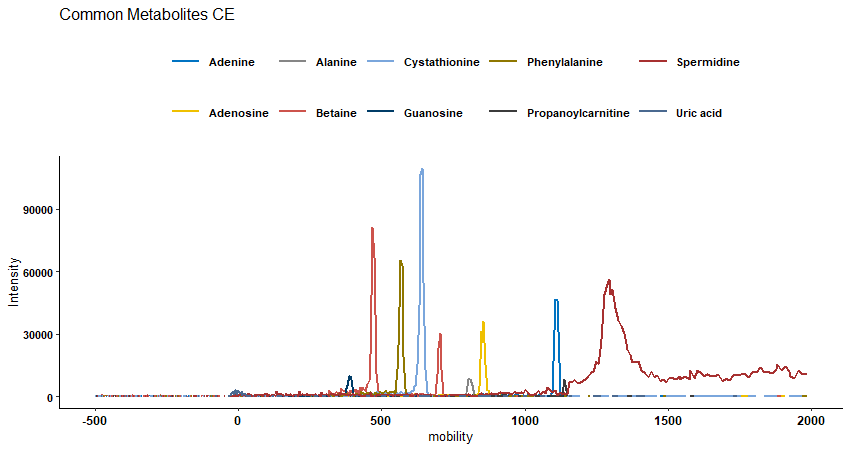

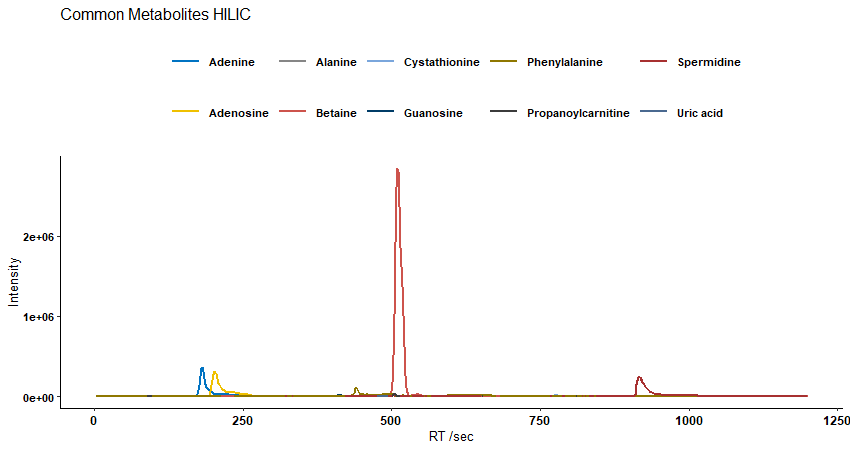


*Figure S5* Separation of a selection of commonly detected metabolites in QC sample
